# Association of C‐reactive protein gene polymorphisms with the risk of ischemic stroke: A systematic review and meta‐analysis

**DOI:** 10.1002/brb3.2976

**Published:** 2023-05-23

**Authors:** Wei Chen, Xiaomin Zhu, Yueqiang Hu, Huangzhong Hong, Longjiao Kuang, Ni Liang, Jianmin Zhu, Lingfei Jiang, Lin Wu

**Affiliations:** ^1^ Graduate School of Jiangxi University of Chinese Medicine Nanchang China; ^2^ District 1, Department of Encephalopathy The First Affiliated Hospital of Guangxi University of Chinese Medicine Nanning China; ^3^ The First Clinical Faculty of Guangxi University of Chinese Medicine Nanning China; ^4^ Graduate School of Guangxi University of Chinese Medicine Nanning China; ^5^ Guangxi Key Laboratory of Basic Research of Traditional Chinese Medicine Nanning China; ^6^ Guangxi Scientific Research Center of Traditional Chinese Medicine Nanning China

**Keywords:** case‐control study, C‐reactive protein, ischemic stroke, Meta‐analysis, Polymorphism

## Abstract

**Background and purpose:**

The heterogeneous, complex condition known as ischemic stroke (IS) is brought on by the interaction of a number of risk factors and genetic variables. The association between C‐reactive protein (CRP) gene polymorphisms and IS has, however, been the subject of inconsistent findings. Therefore, we conducted a meta‐analysis to comprehensively address possible associations of CRP genes with the risk of IS.

**Methods:**

A comprehensive literature search for all the published articles was performed in electronic databases including PubMed, EMBASE, Cochrane Library, and Google Scholar from January 1, 1950 to June 30, 2022. Odds ratio (OR) with 95% Confidence interval (CIs) along with fixed/random effect models were used to calculate summary estimates.

**Results:**

Twelve case‐control studies totalling 3880 IS cases and 5233 controls were included for the association of CRP gene polymorphisms (rs1800947, rs1130864, rs3093059, rs2794521, and rs1205). Across all genotyping models, we discovered that rs1130864, rs3093059, rs2794521, and rs1205SNPs were not substantially related to IS risk. A trend for significant association for rs1800947 under dominant (OR = 1.19; 95% CI = 0.97 to 1.48), recessive (OR = 1.49; 95% CI = 0.71 to 3.14) and allelic model (OR = 1.21; 95% CI = 0.99 to 1.48) was observed. However, protective association for rs1130864 under dominant (OR = 0.80; 95% CI = 0.70 to 0.91) and rs3093059 under allelic model (OR = 0.18; 95% CI = 0.14 to 0.22) was found.

**Conclusion:**

Our thorough study revealed that the CRP gene variants rs1800947, rs1130864, rs3093059, rs2794521, and rs1205 could not be related to the risk of ischemic stroke. However, additional research must focus on the rs1800947 polymorphisms in a particular group.

## Graphical abstract

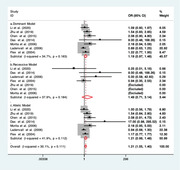

## 1 INTRODUCTION

The second most prevalent cause of mortality worldwide is a stroke. (Feigin et al., 2014) About 80% of all strokes are ischemic strokes, which are a diverse, complex condition brought on by one or more risk factors such age, hypertension, diabetes mellitus, hyperlipemia, smoking, and obesity. (Nath et al., 2022) These risk factors, however, only account for a portion of the condition. (Appunni et al., 2022; Flossmann et al., 2004) Animal models have revealed the significant genetic component of stroke in twin and family‐based association studies. (Boehme et al., 2017) Atherosclerosis, a significant contributor to ischemic stroke, is mostly mediated by inflammation. (Anrather & Iadecola, 2016) C‐Reactive protein (CRP) is a crucial inflammatory measure that plays a significant part in determining the prognosis of IS. (VanGilder et al., 2014; Yu et al., 2019)

According to reports, elevated serum CRP levels enhance the risk of IS (Di Napoli et al., 2005; Mengozzi et al., 2020; Ridker et al., 1995; Smith et al., 2006). The genetic variants within the CRP gene, which is present on chromosomes 1q21 to 1q23, should be considered a key contributor to the altered serum level of CRP. CRP genotype may more precisely reflect the baseline and long‐term of exposure to CRP than blood CRP levels, which only indicate the level of inflammation at a specific time point. However, the association between CRP single nucleotide polymorphisms (SNPs) (rs1800947, rs1130864, rs3093059, rs2794521, and rs1205) and ischemic stroke is still debatable. SNPs 2667 [rs1800947] is located in exon 2; 3872 [rs1205] located in the 3‐flanking region at position +1846; SNP 790 (rs3093058) located at position −757 in the 5‐promoter region of CRP gene (Hage & Szalai, 2007). It is well known that the rs1130864 variant is found in the 3′ untranslated region at position +1444 of the CRP gene and that its predominant allele has been associated with lower levels of circulating CRP. (Komurcu‐Bayrak et al., 2009; Kong et al., 2012; Miller & Zee et al., 2005; Ridker et al., 1998)

The correlations between IS and variations in the eNOS, ACE, ANRIL, MTHFR, and IL‐10 genes have been identified in previous meta‐analyses. (Alhazzani et al., 2018; Kumar et al., 2020; Kumar et al., 2017; Rui et al., 2020; Wei et al., 2017) Additionally, a growing body of research on CRP candidate genes has produced some contradictory findings. (Chen et al., 2015; Das et al., 2014; Du et al., 2015 Jun, 3; Flex et al., 2004; Ladenvall et al., 2006; Li et al., 2020; Morita et al., 2006; Shen et al., 2013; Wu et al., 2017; Zhou et al., 2017; Zhu et al., 2019) In order to fully address potential association of CRP genes with the pathogenesis of IS, we set out to conduct a meta‐analysis.

## 2 METHODS

### 2.1 Search strategy

The systematic literature review was performed using the guidelines of the Preferred Reporting Items for Systematic Reviews and Meta‐analyses (PRISMA) statement. (Moher et al., 2015) A comprehensive search for all the published articles was performed in electronic databases including PubMed, EMBASE, Cochrane Library, and Google Scholar from 01^st^ January 1950 to 30^th^ June 2022. The search criteria include the following terms: (“Ischemic stroke” OR “cerebral vascular accident” OR “CVA” OR “atherothrombotic cerebral infarction” OR “cerebral stroke”) AND (“CRP” OR “C Reactive Protein”) AND (“rs1800947”, OR “rs1130864”, “rs3093059”, “rs2794521” OR “rs1205”) AND (“Gene” OR "Genotype). The literature search covered all English‐language articles. Additionally, the reference list of retrieved studies, review articles and previous meta‐analyses, were manually searched for collecting more relevant studies, which were not found while performing the electronic search. Studies involving human subjects were considered in our meta‐analysis. When there were duplicates, the source with the most detailed information was utilised.

### 2.2 Eligibility criteria

Studies that matched the following criteria and focused on the association between CRP gene polymorphism and risk of ischemic stroke were included:

**Inclusion criteria**:

(a) Case‐control studies investigating for the association of CRP gene polymorphisms rs1800947 and/or rs1130864 and/or rs3093059 and/or rs2794521 and/or rs1205 with risk of IS.; (b) The number of controls and cases in each group were reported; and (c) The odds ratio (OR) and 95% confidence interval (CI) or the data that might be used to calculate the OR and CI were used to describe the genotype and allele distribution.

**Exclusion criteria**:

(a) Letters, editorials, case studies, reviews, meta‐analyses, commentary, studies involving animals or the environment, and (b) non‐English studies.

### 2.3 Risk of bias in individual studies

The risk of bias was assessed by New‐castle Ottawa Scale (NOS) for quality assessment of all the included studies in the meta‐analysis. (Stang, 2010) NOS Scale considers participant selection, comparability, and exposure, to assess the case‐control study's quality. Nine points was the maximum score. Studies rated eight or higher were regarded as being of excellent quality. Publication bias was assessed by using the funnel plot analysis. The asymmetry of the funnel plot was determined by using the Begg's and Egger's regression test. (Begg & Mazumdar, 1994; Egger et al., 1997)

### 2.4 Data extraction

We gathered following information: the first author's name, the publication year, the nation, the method for measuring genotype, the sample size, the proportion of male and female participants, the mean age, and the genotype frequencies of the case and control groups. Separately reviewing the publications and gathering data, the two authors settled any disagreements by consensus or with the help of a third author.

### 2.5 Statistical analysis

To determine the strength of the association between CRP gene polymorphisms and risk of IS, Odds Ratio (OR) with 95% confidence interval (CI) was calculated under the dominant model, recessive model, and allele model. Heterogeneity was calculated with the *I* ^2^ statistics. Fixed effect model was used if *I* ^2^ of less than 50% was observed while *I* ^2^ more than 50% is viewed as moderate to considerable heterogeneity and random effect model was used. A sensitivity analysis was performed by sequentially omitting a single study in each turn, to validate the pooled observed effect. All statistical analyses were done by STATA version 13.1 software.

### 2.6 Trial sequential analysis

Trial sequential analysis (TSA) was conducted by following the guidelines mentioned by Meng et al. (Meng et al., 2018) To determine the necessary sample size, we chose a type I error significance of 5%. We then constructed the TSA monitoring boundaries using TSA software Version 0.9.5.10 Beta.

## 3 RESULTS

**FIGURE 1:**
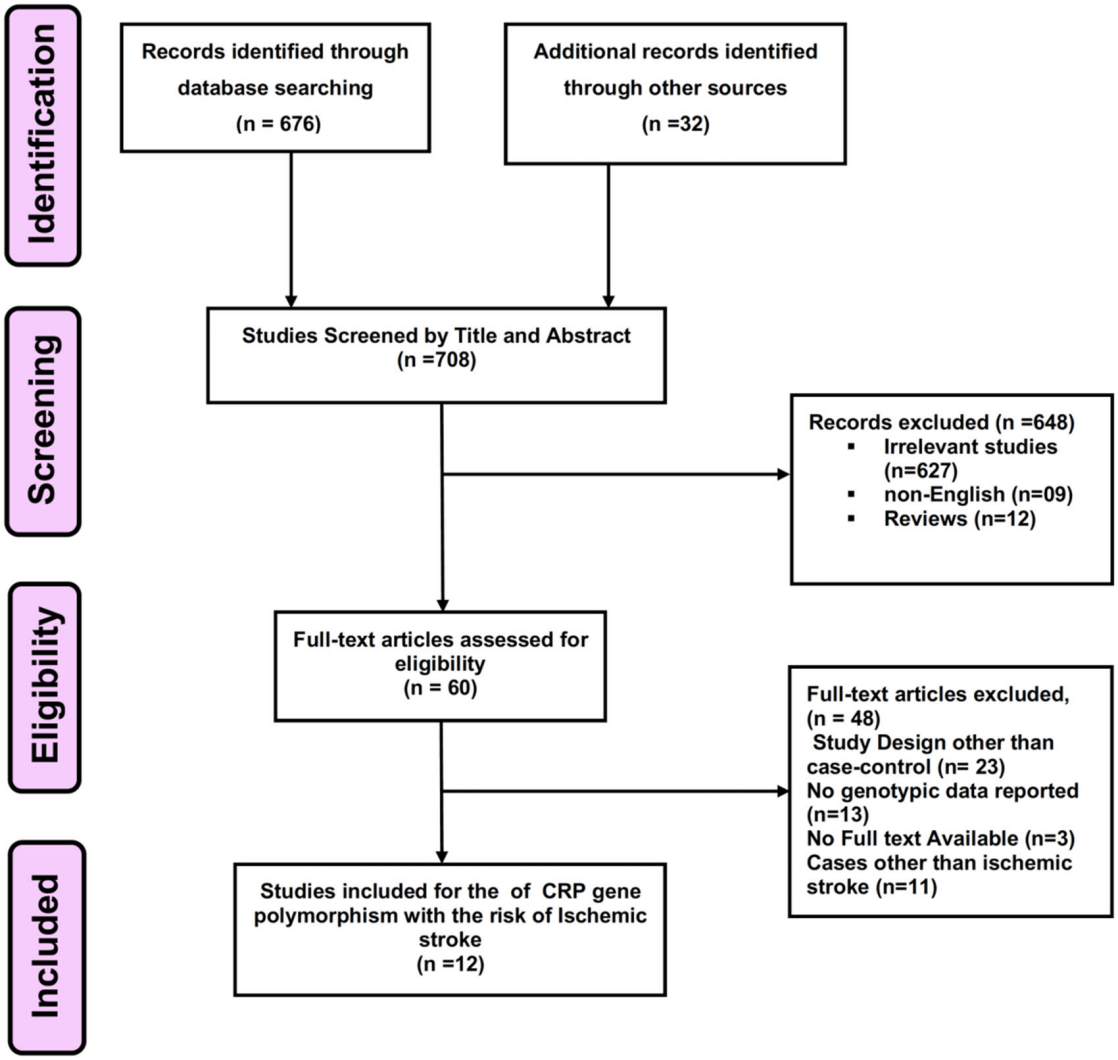
Flow diagram for the selection of studies and specific reasons for exclusion from the present meta‐analysis.

Figure 1 depicts the schematic representation of the PRISMA flowchart for the selection and inclusion of the studies. Through the use of the databases in PubMed, Web of Science, and EMbase, 708 records were found in total, but 648 of those records were excluded due to duplication, unrelated articles, letters, comments, reports, meeting abstracts, a lack of full‐text and insufficient data, and other SNPs populations. Sixty full‐text articles were evaluated for their eligibility out of which 12 case‐control studies (Andersson et al., 2009; Chen et al., 2015; Das et al., 2014; Du et al., 2015; Flex et al., 2004; Ladenvall et al., 2006; Li et al., 2020; Morita et al., 2006; Shen et al., 2013; Wang et al., 2009; Wu et al., 2017; Zhu et al., 2019) were finally included in our systematic review and meta‐analysis.

### 3.1 Characteristics of eligible studies

Table 1 summarizes the key baseline characteristics of the literature analyzed in this thorough research. A total of 12 case‐control studies (Andersson et al., 2009; Chen et al., 2015; Das et al., 2014; Du et al., 2015 Jun, 3; Flex et al., 2004; Ladenvall et al., 2006; Li et al., 2020; Morita et al., 2006; Shen et al., 2013; Wang et al., 2009; Wu et al., 2017; Zhu et al., 2019) were included in which the association between the rs1800947 and IS risk were reported in seven studies, (Chen et al., 2015; Das et al., 2014; Flex et al., 2004; Ladenvall et al., 2006; Li et al., 2020; Morita et al., 2006; Zhu et al., 2019) rs1130864 in five studies, (Andersson et al., 2009; Du et al., 2015 Jun, 3; Ladenvall et al., 2006; Li et al., 2020; Morita et al., 2006) rs3093059 in five studies, (Chen et al., 2015; Du et al., 2015 Jun, 3; Li et al., 2020; Shen et al., 2013; Wu et al., 2017) rs2794521 in four studies, (Ladenvall et al., 2006; Shen et al., 2013; Wang et al., 2009; Wu et al., 2017), and rs1205 in three studies (Morita et al., 2006; Wang et al., 2009; Wu et al., 2017) respectively. Seven included studies (58.3%) were from China. The remaining other five studies were from India, Japan, Italy, and two from Sweden. A total of 3880 cases and 5233 controls were included involving 12 studies. The sample size ranged from 138 to 993 and the year of publication were from 2004 to 2020. All included studies used Taqman, sequencing, and polymerase chain reaction‐restriction fragment length polymorphism (PCR/RFLP) as their genotyping methods and the sources of samples were whole blood for detecting the polymorphism. The NOS scores varied from 7 to 9, and 67% (8/12) of the studies were of excellent quality, indicating that the methodology was adequate. Table S1 is the PRISMA 2020 checklist used to guide the reporting of this systematic review and meta‐analysis. The PRISMA checklist is a widely recognized tool for ensuring the completeness and transparency of reporting in systematic reviews and meta‐analyses. The checklist includes 27 items covering the different aspects of a systematic review and meta‐analysis, such as the study design, search strategy, study selection process, data extraction, and risk of bias assessment. We used this checklist to guide our reporting and ensure that we have included all relevant information in this manuscript. Please refer to Table S1 for the full PRISMA checklist. Additionally, Table 2 provided a detailed summary of the included studies' genotype frequencies.

**TABLE 1:** Characteristic of the included studies in the meta‐analysis for the between CRP gene polymorphism with the risk of ischemic stroke.

S. No	Author Name & Year	Country	Total IS Cases	Total Control	M/F Cases	M/F Control	Mean Age Cases	Mean Age Controls	Variant Studied	Genotype method	NOS Quality score
1.	Li et al. (2020)	China	236	291	158/78	167/124	67.14(9.69)	66.56(6.58)	rs1130864, rs1800947 and rs3093059	PCR‐RFLP	7
2.	Zhu et al. (2019)	China	138	276	69/69	138/138	60(55,64)	60 (54,63)	rs1800947	Sequencing	8
3.	Wu et al. (2017)	China	580	582	320/260	332/250	65.8(15.9)	67.5(16.2)	rs3093059, rs2794521 and rs1205	TaqMan	9
4.	Chen et al. (2015)	China	159	175	112/47	106/69	65.45(8.15)	65.75(8.65)	rs1800947 and rs3093059	PCR‐RFLP	7
5.	Du et al. (2015)	China	158	290	110/48	172/118	67.98(7.73)	66.77(10.72)	rs1130864 and rs3093059	PCR–RFLP	7
6.	Das et al. (2014)	India	200	200	168/32	168/32	49.4(17.4)	49.08(16.9)	rs1800947	PCR‐RFLP	8
7.	Shen et al. (2013)	China	548	993	270/278	399/594	65.48(11.76)	64.97(9.16)	rs3091244, rs2794521, rs876537 and rs3093059	PCR‐RFLP	9
8.	Anderson et al. (2009)	Sweden	308	735	175/133	436/299	54.9(8.2)	54.8(8.2)	rs1130864	Taqman	9
9.	Wang et al. (2009)	China	564	564	353/211	364/200	61.01(0.42)	62.23(0.39)	rs3091244, rs2794521, rs1205	TaqMan	7
10.	Morita et al. (2006)	Japan	152	304	87/65	149/155	72.4(8.2)	78.0(4.2)	rs1130864, rs1800947, rs1341665 and rs1205	Taqman	8
11.	Ladenvall et al. (2006)	Sweden	600	600	385/215	385/215	56(10)	56(10)	rs2794521, rs3091244, rs1800947 and rs1130864	Taqman	9
12.	Flex et al. (2004)	Italy	237	223	132/105	107/116	76.2(9.4)	76.1(6.8)	rs1800947	PCR‐RFLP	8

Abbreviations: IS‐ Ischemic Stroke; NOS‐New‐Castle Ottawa Scale.; PCR‐RFLP‐Polymerase Chain Reaction‐Restriction Fragment Length Polymorphism; SNP‐ Single Nucleotide Polymorphism.

**TABLE 2:** Genotypic distribution among cases and controls for all the included studies.

S. No	Author Name & Year	Sample size Cases/Controls	Cases			Controls			Allele (Major/Minor)	MAF	p‐value
			Mutant	Heterozygous	Wild	Mutant	Heterozygous	Wild			
**rs1800947**											
1.	Li et al. (2020)	236/291	0	22	214	2	23	266	G/C	0.046	0.069
2.	Zhu et al. (2019)	138/276	0	20	118	0	26	250	G/C	0.047	0.412
3.	Chen et al. (2015)	159/175	0	17	142	0	9	166	G/C	0.026	0.727
4.	Das et al. (2014)	200/200	4	0	196	0	0	200	G/C	0.000	–
5.	Morita et al. (2006)	152/304	0	12	140	0	11	293	G/C	0.018	0.748
6.	Ladenvall et al. (2006)	600/600	5	66	528	1	79	520	G/C	0.068	0.261
7.	Flex et al. (2004)	237/223	8	40	189	8	29	186	G/C	0.101	<0.001
**rs1130864**											
8.	Li et al. (2020)	236/291	0	29	207	0	42	249	C/T	0.072	0.185
9.	Du et al. (2015)	158/290	0	16	142	0	42	248	C/T	0.072	0.184
10.	Anderson et al. (2009)	308/735	17	111	139	46	214	284	C/T	0.281	0.52
11.	Morita et al. (2006)	152/304	1	19	132	1	38	265	C/T	0.066	0.768
12.	Ladenvall et al. (2006)	600/600	61	258	281	56	257	287	C/T	0.308	0.888
**rs3093059**											
13.	Li et al. (2020)	236/291	40	85	111	33	103	155	T/C	0.290	0.016
14.	Wu et al. (2017)	580/582	26	172	382	43	238	301	A/G	0.278	0.666
15.	Chen et al. (2015)	159/175	3	48	108	4	56	115	T/C	0.183	0.349
16.	Du et al. (2015)	158/290	5	52	101	4	86	200	T/C	0.162	0.118
17.	Shen et al. (2013)	548/993	13	148	386	31	314	649	T/C	0.189	0.346
**rs2794521**											
18.	Wu et al. (2017)	580/582	27	183	370	56	234	292	A/G	0.297	0.364
19.	Shen et al. (2013)	548/993	14	147	385	33	270	691	T/C	0.169	0.298
20.	Wang et al. (2009)	564/564	23	155	386	11	155	398	A/G	0.157	0.358
21.	Ladenvall et al. (2006)	600/600	45	261	293	45	261	294	T/C	0.293	0.211
**rs1205**											
22.	Wu et al. (2017)	580/582	30	172	378	53	222	307	T/C	0.282	0.165
23.	Wang et al. (2009)	564/564	110	282	172	94	297	173	T/C	0.430	0.078
24.	Morita et al. (2006)	152/304	12	68	72	42	125	137	G/A	0.344	0.122

### 3.2 Association between CRP (rs1800947) gene polymorphism and IS risk

**FIGURE 2:**
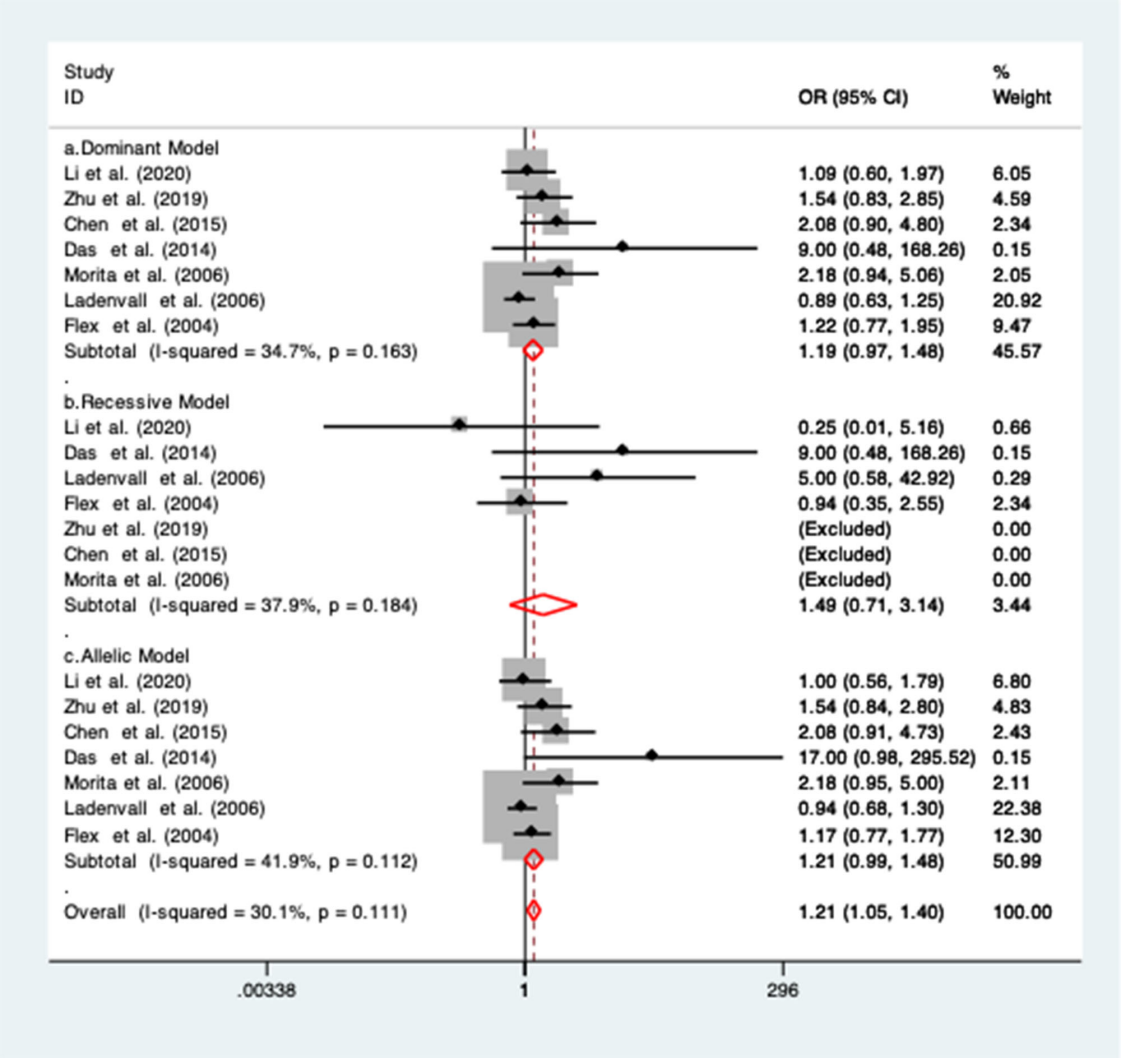
Forest plots of the association between rs1800947 SNP with ischemic stroke risk under (a) dominant model; (b) recessive model; and (c) allelic model.

Seven studies (Chen et al., 2015; Das et al., 2014; Flex et al., 2004; Ladenvall et al., 2006; Li et al., 2020; Morita et al., 2006; Zhu et al., 2019) were included for the association between rs1800947 gene polymorphisms and IS risk. Across all genotyping models, a trend for the significant association for rs1800947 under dominant (OR = 1.19; 95% CI = 0.97 to 1.48; *I* ^2^ = 34.7%), recessive (OR = 1.49; 95% CI = 0.71 to 3.14; *I* ^2^ = 37.9%] and allelic model (OR = 1.21; 95% CI = 0.99 to 1.48; *I* ^2^ = 41.9%) was observed (Figure 2). A fixed‐effect model was applied to pool the data from the included studies as a lower degree of heterogeneity was observed. The summary estimates for the association between CRP gene polymorphism with the risk of ischemic stroke are represented in Table 3.

**TABLE 3:** Summary estimates for the between CRP gene polymorphism with the risk of ischemic stroke.

rsID	No. of Studies	Genetic Model	Pooled Estimates		Heterogeneity	
			OR	95 % CI	I (Nath et al., 2022) (%)	*p*‐Value
**rs1800947**	07	Dominant	1.19	0.97 to 1.48	34.7	0.16
		Recessive	1.49	0.71 to 3.14	37.9	0.18
		Allelic	1.21	0.99 to 1.48	41.9	0.11
**rs1130864**	05	Dominant	**0.80**	**0.70 to 0.91**	0	0.80
		Recessive	1.03	0.75 to 1.44	0	0.74
		Allelic	1.03	0.91 to 1.16	0	0.56
**rs3093059**	05	Dominant	0.91	0.75 to 1.11	55.4	0.06
		Recessive	0.98	0.60 to 1.58	53	0.07
		Allelic	**0.18**	**0.14 to 0.22**	83.2	<0.001
**rs2794521**	04	Dominant	0.93	0.79 to 1.10	58.2	0.06
		Recessive	0.90	0.52 to 1.56	74.9	0.008
		Allelic	0.92	0.75 to 1.14	78.9	0.003
**rs1205**	03	Dominant	0.89	0.72 to 1.09	58.2	0.09
		Recessive	0.76	0.44 to 1.31	76.5	0.01
		Allelic	0.87	0.67 to 1.12	79.1	0.008

Abbreviations: CI, confidence interval.; OR, Odds ratio.

Bold values of OR represent statistically significant results (*p*‐value < 0.05).

### 3.3 Association between CRP (rs1130864) gene polymorphism and IS risk

**FIGURE 3:**
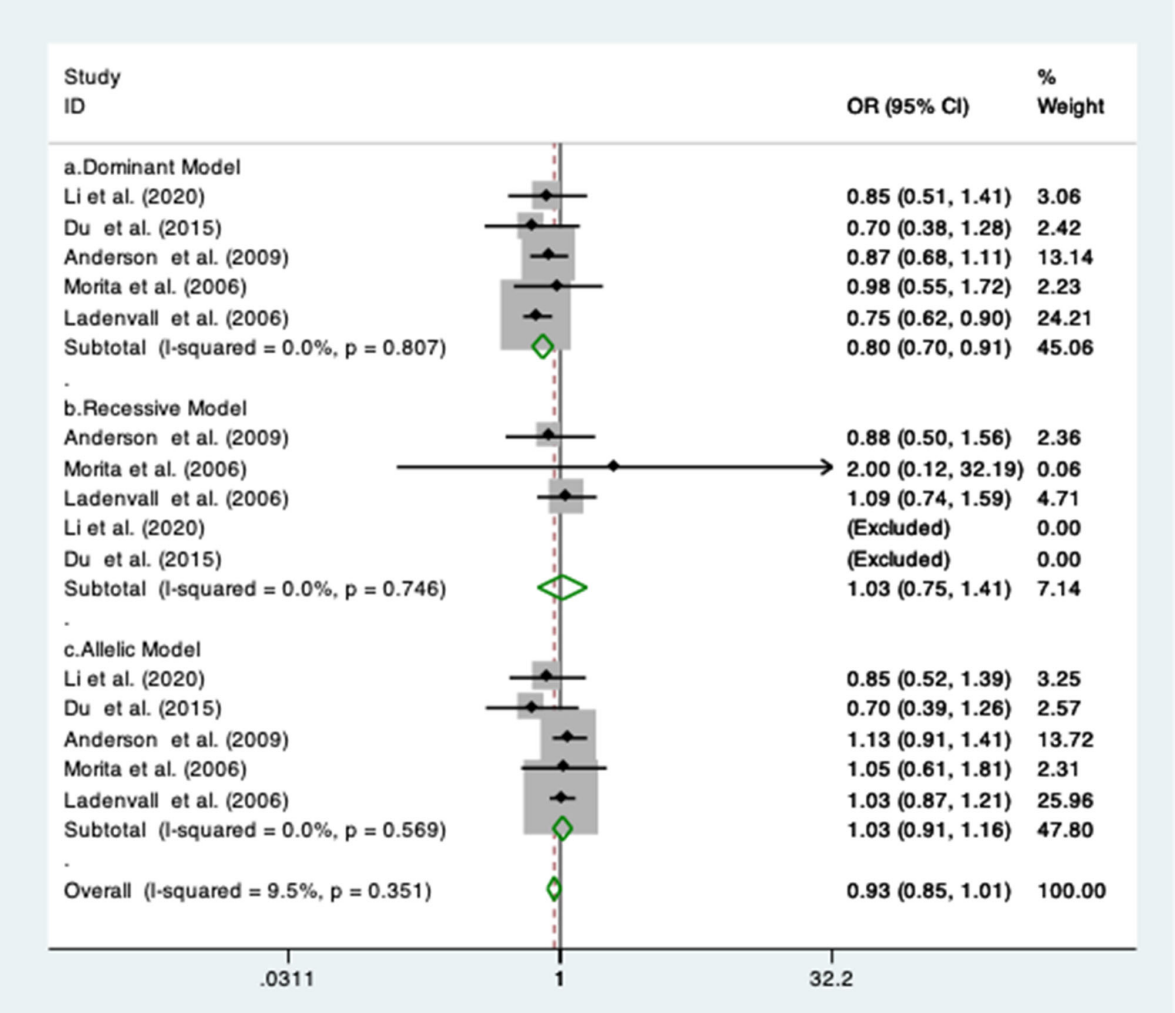
Forest plots of the association between rs1130864 SNP with ischemic stroke risk under (a) dominant model; (b) recessive model; and (c) allelic model.

Five studies (Andersson et al., 2009; Du et al., 2015 Jun, 3; Ladenvall et al., 2006; Li et al., 2020; Morita et al., 2006) reported the data for the association between rs1130864 gene polymorphisms and IS risk. A protective nature of the association between rs1130864 gene polymorphisms and IS risk only under dominant model (OR = 0.80; 95% CI = 0.70 to 0.91; *I* ^2^ = 0%) was observed. No significant association was found under recessive (OR = 1.03; 95% CI = 0.75 to 1.41; *I* ^2^ = 0%) and allelic model (OR = 1.03; 95% CI = 0.91 to 1.16; *I* ^2^ = 0%). The forest plot for all the genetic models is illustrated in Figure 3.

### 3.4 Association between CRP (rs3093059) gene polymorphism and IS risk

**FIGURE 4:**
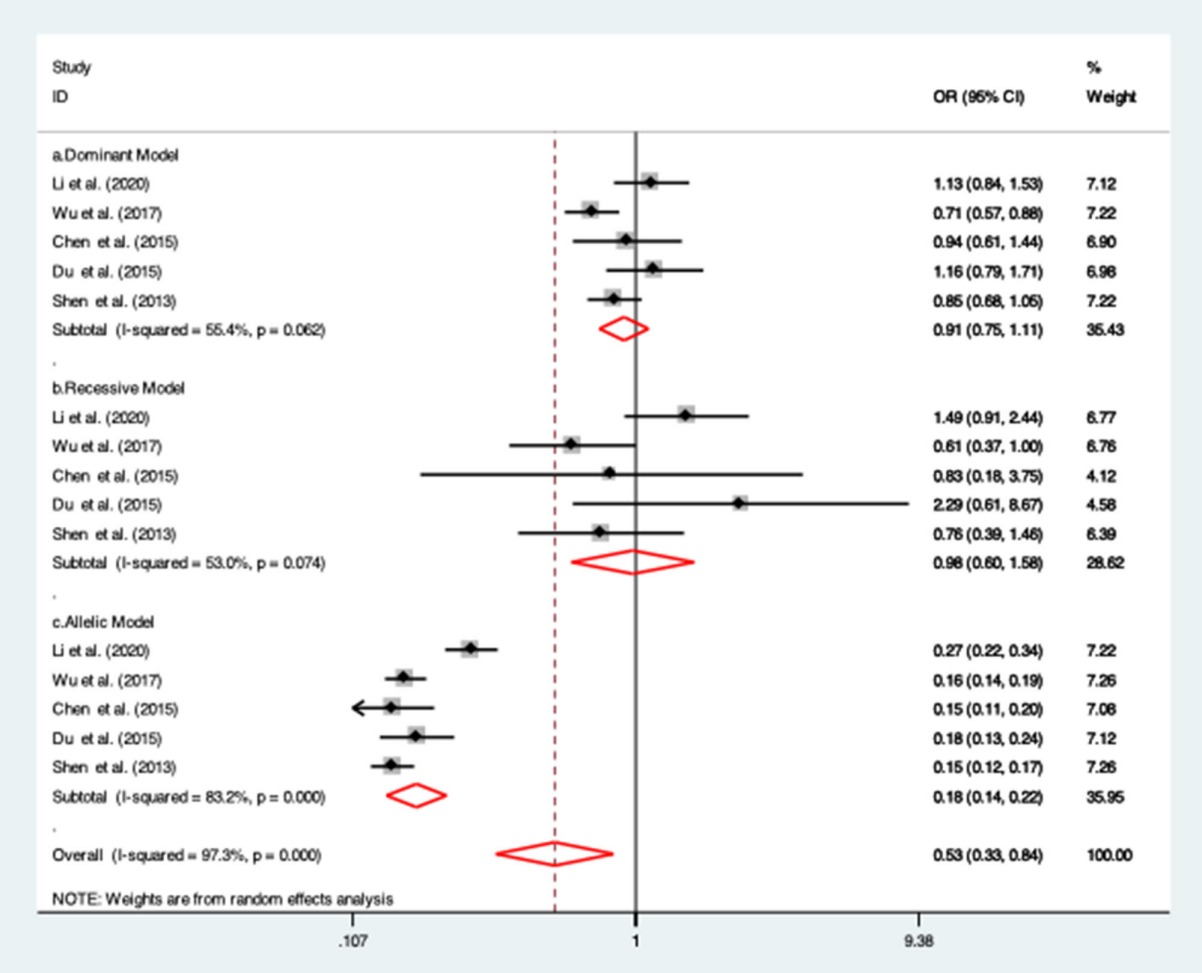
Forest plots of the association between rs3093059 SNP with ischemic stroke risk under (a) dominant model; (b) recessive model; and (c) allelic model.

The association between rs3093059 gene polymorphisms and IS risk was investigated in five studies. (Chen et al., 2015; Du et al., 2015 Jun, 3; Li et al., 2020; Shen et al., 2013; Wu et al., 2017) A protective nature of association between rs3093059 gene polymorphisms and IS risk only under allelic model (OR = 0.18; 95% CI = 0.14 to 0.22; *I* ^2^ = 83.2%) was found. However, non‐significant association was observed under dominant (OR = 0.91; 95% CI = 0.75 to 1.11; *I* ^2^ = 55.4%) and recessive model (OR = 0.98; 95% CI = 0.60 to 1.58; *I* ^2^ = 53%). Due to a high degree of heterogeneity, a random‐effect model was applied (Figure 4).

### 3.5 Association between CRP (rs2794521) gene polymorphism and IS risk

**FIGURE 5:**
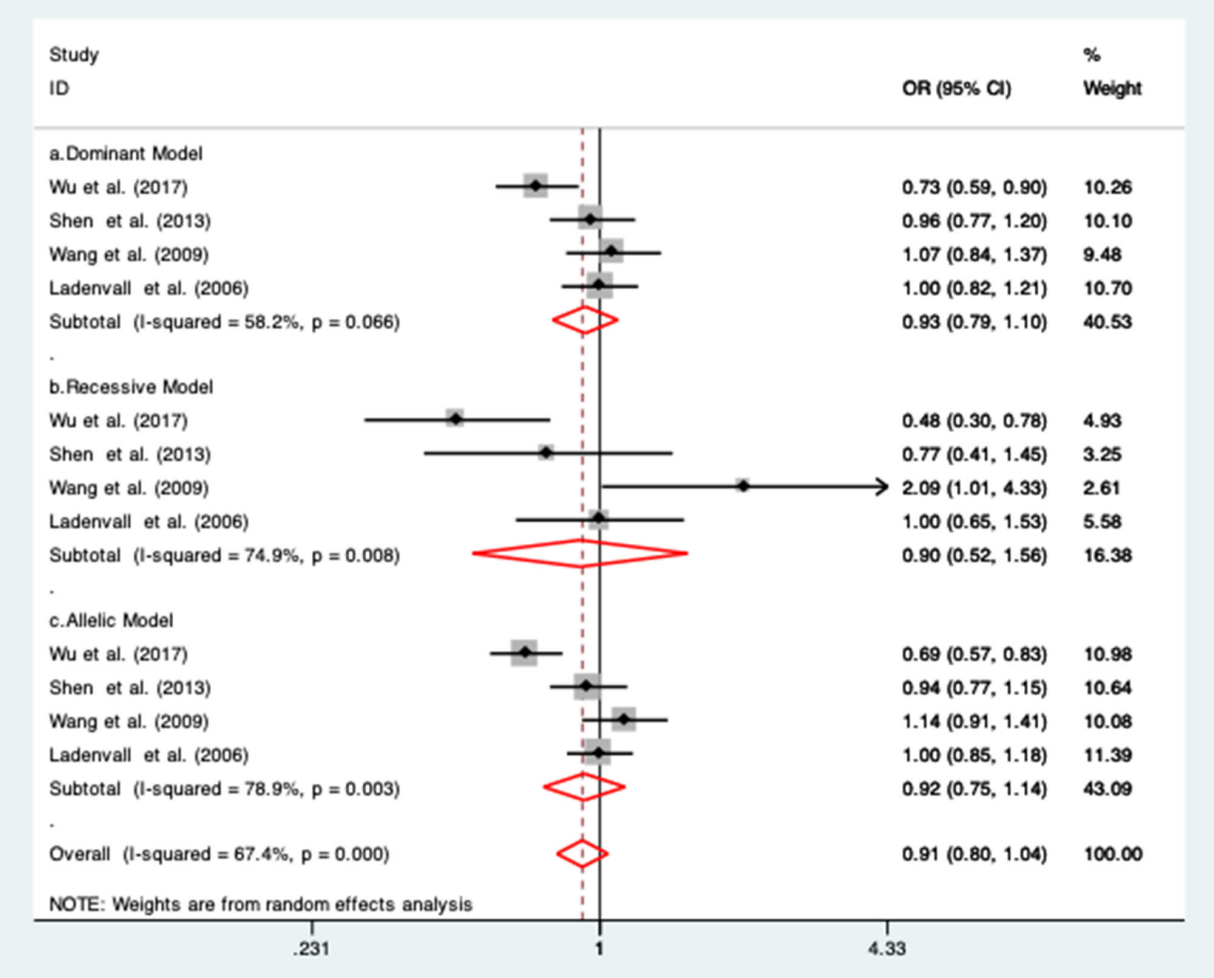
Forest plots of the association between rs279452 SNP with ischemic stroke risk under (a) dominant model; (b) recessive model; and (c) allelic model.

Non‐significant association between rs2794521 gene polymorphisms and IS risk was observed under dominant (OR = 0.93; 95% CI = 0.79 to 1.10; *I* ^2^ = 58.2%), recessive model (OR = 0.90; 95% CI = 0.52 to 1.56; *I* ^2^ = 74.9%) and allelic model (OR = 0.92; 95% CI = 0.75 to 1.14; *I* ^2^ = 78.9%) for all the four included studies (Ladenvall et al., 2006; Shen et al., 2013; Wang et al., 2009; Wu et al., 2017). Random‐effect model was applied as the observed *I* ^2^ was more than 50% (Figure 5).

### 3.6 Association between CRP (rs1205) gene polymorphism and IS risk

**FIGURE 6:**
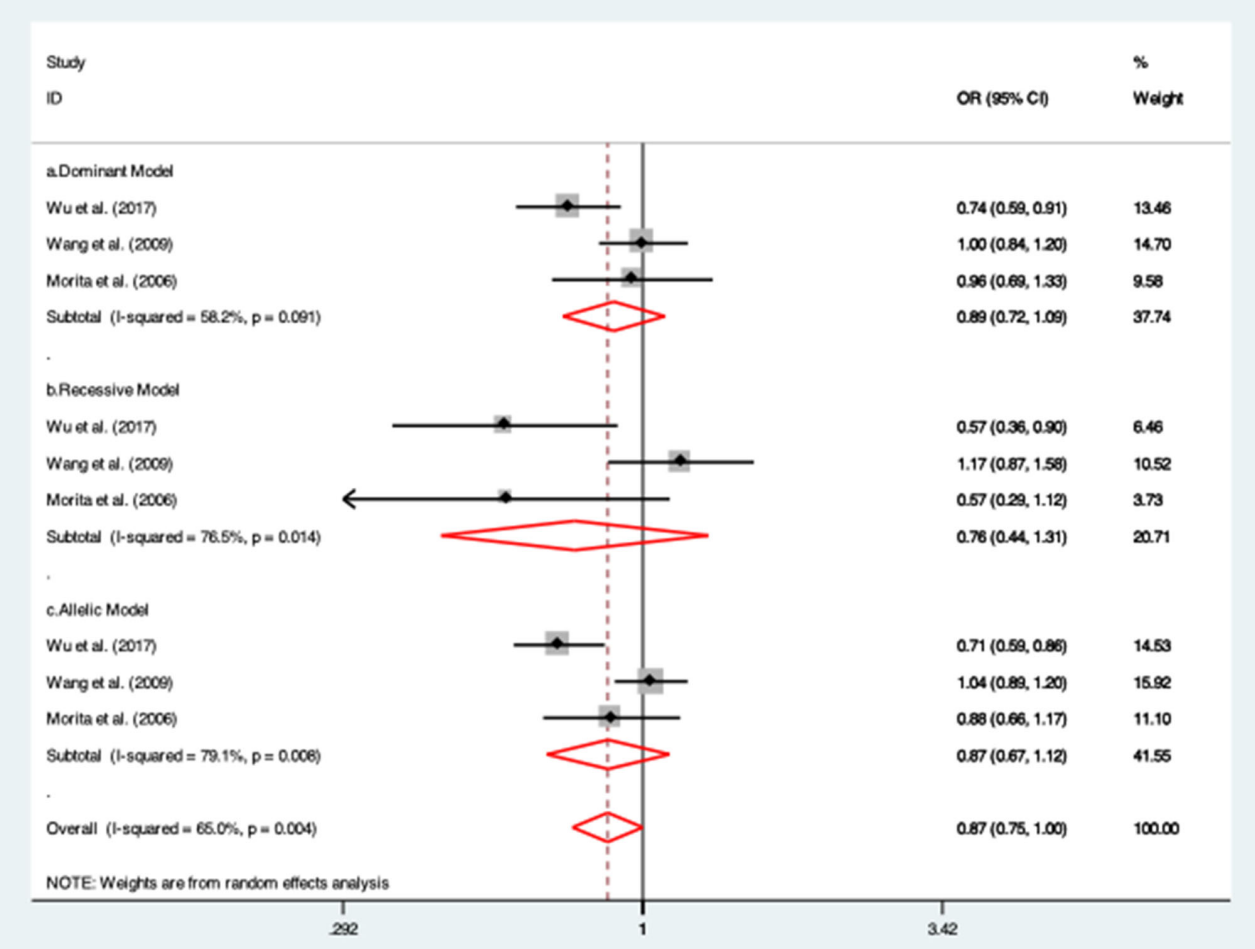
Forest plots of the association between rs1205 SNP with ischemic stroke risk under (a) dominant model; (b) recessive model; and (c) allelic model.

Only three studies (Morita et al., 2006; Wang et al., 2009; Wu et al., 2017) reported the genotypic frequencies for rs1205 SNP. We detected non‐significant association between rs1205 gene polymorphisms and IS risk under dominant (OR = 0.89; 95% CI = 0.72 to 1.09; *I* ^2^ = 58.2%), recessive model (OR = 0.76; 95% CI = 0.44 to 1.31; *I* ^2^ = 76.5%) and allelic model (OR = 0.87; 95% CI = 0.67 to 1.12; *I* ^2^ = 79.1%; Figure 6).

### 3.7 Trial sequential analysis

**FIGURE 7:**
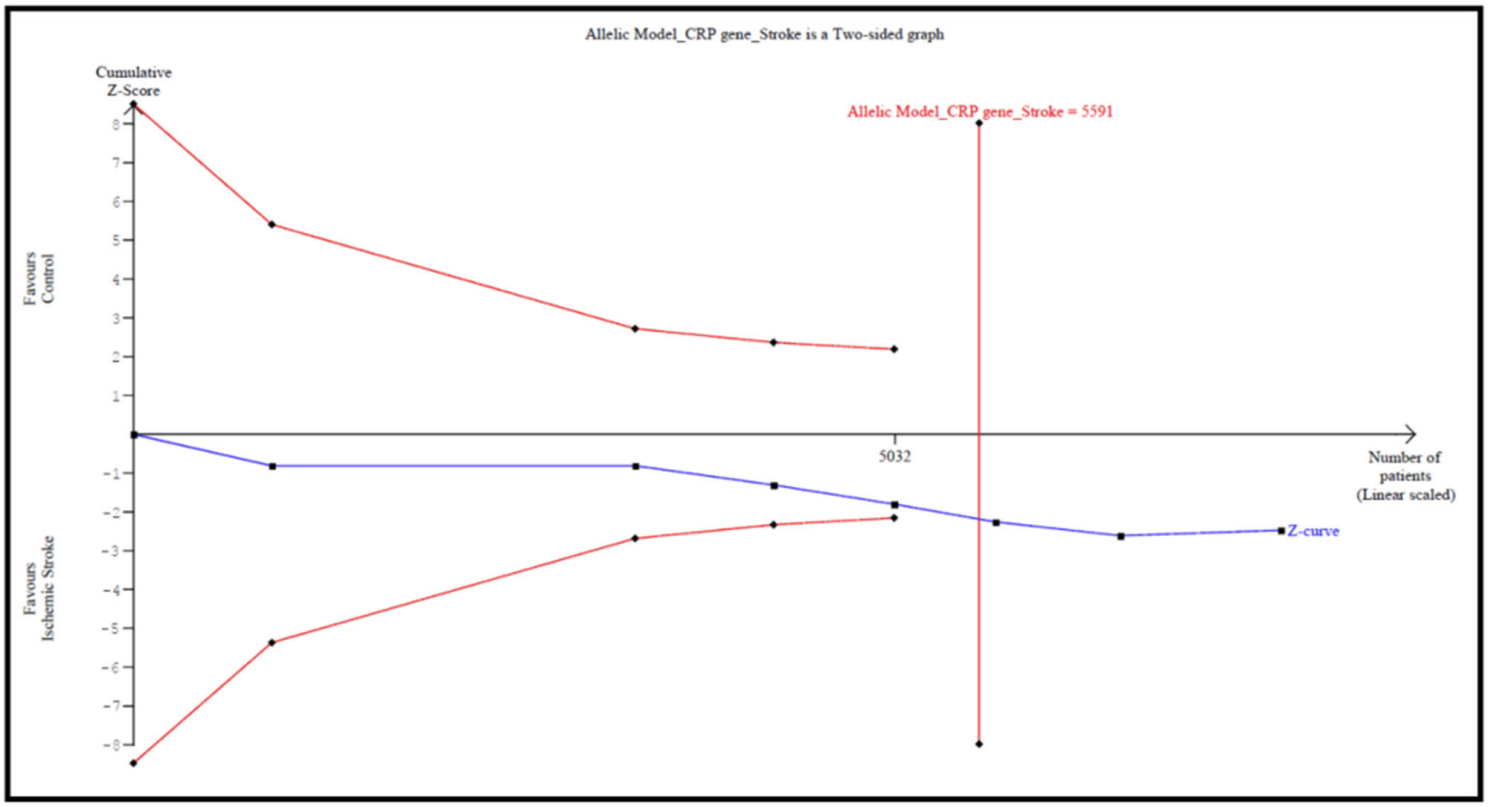
Trial sequential analysis for CRP rs1800947 polymorphism under the allelic model.

The results of TSA are shown in Figure 7, the required sample size is 21,728 samples, and the cumulative z‐curve crossed the trial sequential monitoring boundary before reaching the required sample size, which means that our conclusions are robust with this sufficient evidence.

### 3.8 Sensitivity analyses

Furthermore, we performed sensitivity analyses to assess the influence of each individual study on the pooled ORs by sequential omission of individual included studies. However, the corresponding pooled ORs were not significantly altered by removing any of the studies (Figure S1a–e). Therefore, the sensitivity analysis confirmed that the results of this meta‐analysis were statistically reliable and robust.

### 3.9 Publication bias

Funnel plot and the Egger's test were performed to assess the publication bias arising from the literature included in our meta‐analysis. No obvious asymmetry was observed for the included studies according to the visual assessment of the funnel plot. In addition, there was no statistical evidence of publication bias among the studies using Egger's regression test (*p*‐value: 0.20 for rs1800947; 0.24 for rs1130864; 0.24 for rs3093059; 0.34 for rs2794521; and < 0.12 for rs1205 SNPs; Figure S2a–e).

### 3.10 Trial sequential analysis

The result of TSA is shown in Figure 7, the required sample size is 5591 samples, and the cumulative *z*‐curve does not cross the trial sequential monitoring boundary before reaching the required sample size, which means that more studies with large sample size are required in order to reach to the conclusive findings.

## 4 DISCUSSION

The largest cause of long‐term impairment and mortality worldwide is stroke. Our findings indicate that under the dominant model, recessive model, and allele model, there was a trend for the difference in IS susceptibility and rs1800947 risk was observed. However, protective association for rs1130864 under dominant and rs3093059 under allelic model was found. According to earlier research, changes in plasma CRP levels are biologically significant, and greater CRP levels are linked to a higher risk of IS. (Kaptoge & Di Angelantonio et al., 2010; Zhou et al., 2016) In the current meta‐analysis, we could not discover any association between the IS risk associated with the polymorphisms rs2794521, and rs1205. The 3′ untranslated region of the CRP gene contained the polymorphism rs1130864, which has been discovered to be related to the susceptibility to a number of inflammatory disorders. (Delongui et al., 2017; Schulz et al., 2016) Earlier, the mendelian randomization, however, did not clearly demonstrate that an increased CRP concentration was causally linked to an increased risk of IS. (Zhang et al., 2020)

Additionally, other studies has shown no connection between the rs1130864 polymorphism and the chance of developing IS. (Andersson et al., 2009; Du et al., 2015 Jun, 3; Ladenvall et al., 2006; Li et al., 2020; Morita et al., 2006; Zhang et al., 2020) Ye *et al.* (2018) conducted a genome‐wide association study in Han Chinese patients with IS found that the SNP rs3093059 in the CRP gene influences the 3‐month functional outcome of the first‐ever large artery atherosclerotic stroke. (Ye et al., 2018) Shen et al. (2013) (Shen et al., 2013) in their case‐control study included 993 age‐matched control people and 548 patients with acute IS, revealed that rs3093059 was statistically substantially linked with IS which was consistent with Wu et al. (2017) (Wu et al., 2017) findings.

The connections between CRP gene polymorphisms and IS have been reported in a large number of studies over the last few decades, and conflicting results between different studies have caused concern. Accordingly, four meta‐analyses published by Zhou et al. (2017), González‐Giraldo et al. (2016), Liu et al. (2015), and Chen et al. (2014), respectively, reported the connection between CRP gene polymorphisms and IS susceptibility. One of which showed no significant associations between these two polymorphisms (rs1800947 and rs1205) in CRP genes and IS (González‐Giraldo et al., 2016), one showed scant evidence to support a role for CRP gene rs2794521 polymorphisms in ischemic stroke predisposition, (Liu et al., 2015) and one could provide the first proof that genetic variants (rs1130864 and rs2794521) within the CRP locus are associated with ischemic stroke. (Chen et al., 2014)

According to the best of our knowledge, this is the first thorough meta‐analysis and systematic review analyzing the association of five SNPs in the CRP gene with IS. Although conducted comprehensively, following limitations were present in our meta‐analysis: (i) certain case‐control studies did not pass the Hardy–Weinberg equilibrium test, suggesting that there was a bias in the selection of the control group; (ii) the studies included in our systematic review and meta‐analysis varied in terms of ethnicity, age and environmental factors and sources of control subjects; (iii) we did not check for the false discovery rate which might arise from the multiple comparisons made in the meta‐analysis; (iv) due to the fact that among matched groups, the other covariates corrected were not the same, whereas within unmatched groups, some studies may have had other covariates adjusted for them while others may have had none at all and lastly; and (v) significant heterogeneity was present in some of the comparisons made in our meta‐analysis. However, we used a random‐effect model throughout to account for the between study heterogeneity and also assessed the quality of each included study.

## 5 CONCLUSION

Our thorough study revealed that the CRP gene variants rs1800947, rs1130864, rs3093059, rs2794521, and rs1205 could not be related to the risk of ischemic stroke. However, additional research must focus on the rs1800947 polymorphisms in a particular group.

## AUTHOR CONTRIBUTIONS

All authors contributed to the study conception and design. W.C., X.Z., L.J., and L.W. independently assessed the quality of included studies. Y.H., H.H., L.K., N.L., and J.Z. performed material preparation, data collection, and analysis. W.C., X.Z., L.J., and L.W. did the analysis of results. All authors read and approved the final draft.

## CONFLICT OF INTEREST STATEMENT

The authors report no relevant conflict of interest.

### PEER REVIEW

The peer review history for this article is available at https://publons.com/publon/10.1002/brb3.2976.

## Supporting information


**Figure‐S1(a‐e)**: Sensitivity Plot for the association between CRP gene polymorphisms (a) rs1800947; (b) rs1130864; (c) rs3093059; (d) rs2794521 and (e)rs1205 with risk of IS.
**Figure‐S2(a‐e)**: Funnel Plot for the association between CRP gene polymorphisms (a) rs1800947; (b) rs1130864; (c) rs3093059; (d) rs2794521 and (e) rs1205 with risk of IS.

**Table S1**: PRISMA checklist 2020

## Data Availability

The data that support the findings of this study are available from the corresponding author upon reasonable request.
