# Supplementary material for: Association of C‐reactive protein gene polymorphisms with the risk of ischemic stroke: A systematic review and meta‐analysis
Source: Brain Behav. 2023 May 23;13(6):e2976. doi: 10.1002/brb3.2976 (PMC10275524; doi:10.1002/brb3.2976)
Supplement: Supplementary file 1 — Figure‐S1(a‐e): Sensitivity Plot for the association between CRP gene polymorphisms (a) rs1800947; (b) rs1130864; (c) rs3093059; (d) rs2794521 and (e)rs1205 with risk of IS. Figure‐S2(a‐e): Funnel Plot for the association between CRP gene polymorphisms (a) rs1800947; (b) rs1130864; (c) rs3093059; (d) rs2794521 and (e) rs1205 with risk of IS. [file BRB3-13-e2976-s001.docx]

**Figure-S1(a-e)**: Sensitivity Plot for the association between CRP gene polymorphisms (a) rs1800947; (b) rs1130864; (c) rs3093059 ; (d) rs2794521 and (e )rs1205 with risk of IS.

1. **rs1800947**


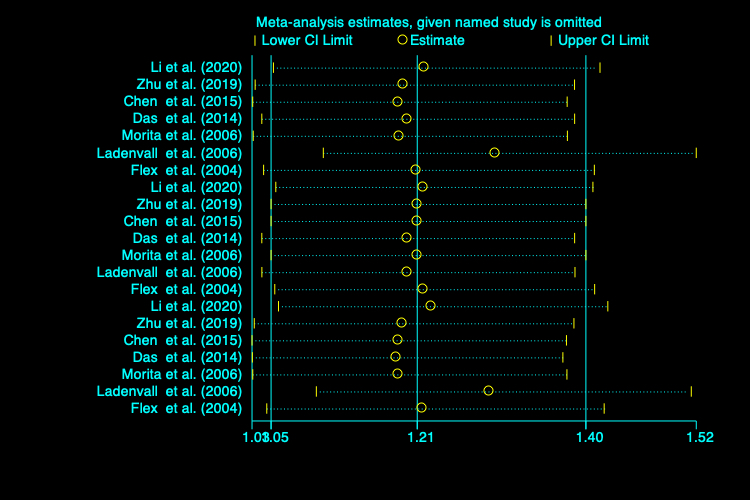


**(b) rs1130864**


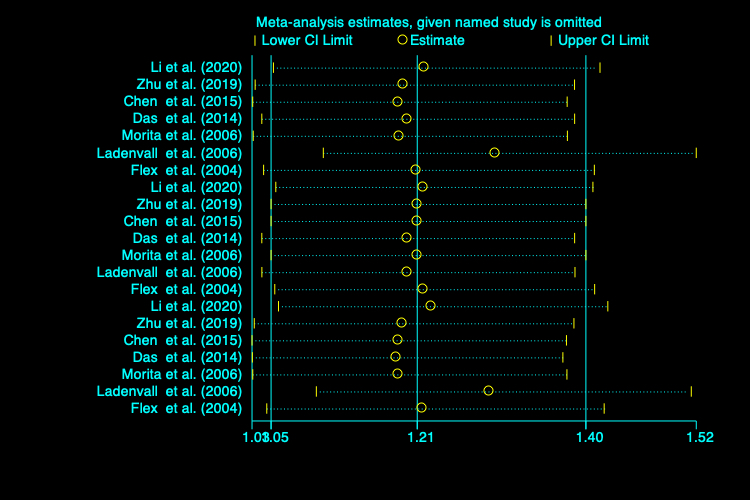


**(c) rs3093059**


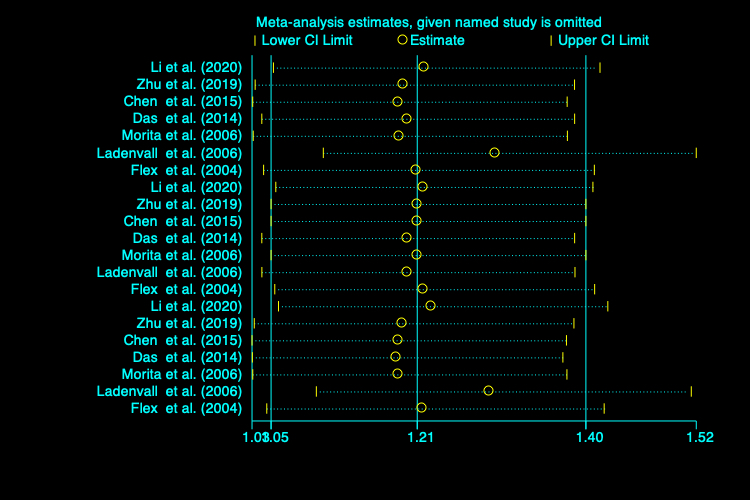


**(d) rs2794521**


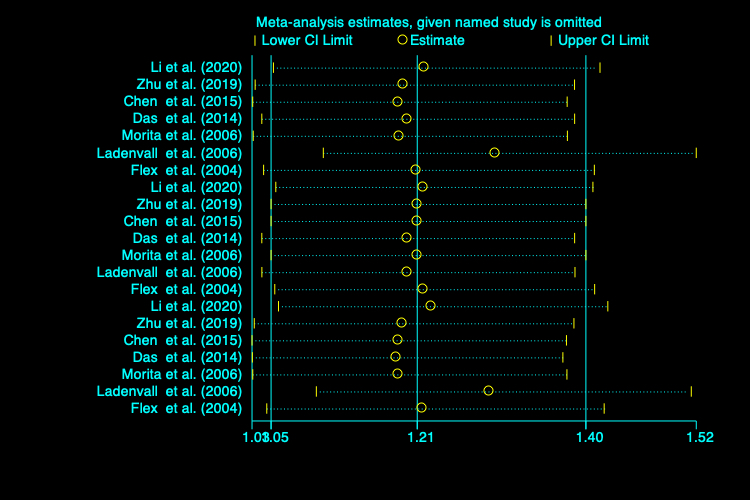


**(e) rs1205**


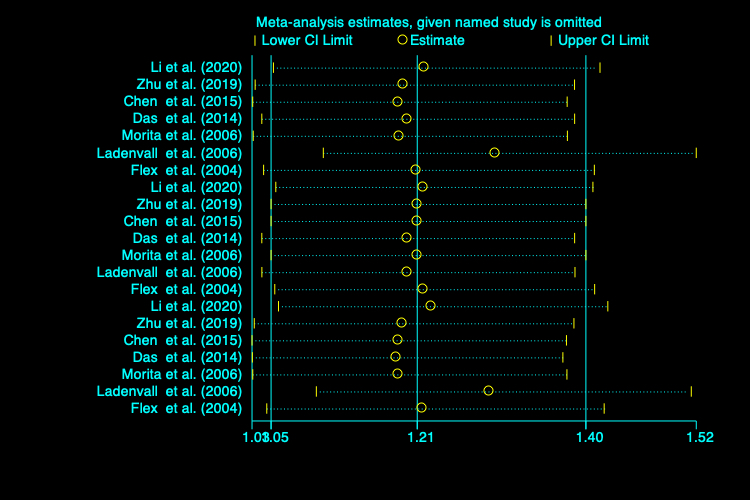


**Figure-S2(a-e)**: Funnel Plot for the association between CRP gene polymorphisms (a) rs1800947; (b) rs1130864; (c) rs3093059 ; (d) rs2794521 and (e) rs1205 with risk of IS.

| **(a) rs1800947**  **** | **(b) rs1130864**  **** |
| --- | --- |
| **(c) rs3093059**  **** | **(d) rs2794521**  **** |
| **(e ) rs1205**  **** | |
